# The integrated analysis of metabolic and protein interaction networks reveals novel molecular organizing principles

**DOI:** 10.1186/1752-0509-2-100

**Published:** 2008-11-25

**Authors:** Pawel Durek, Dirk Walther

**Affiliations:** 1 Bioinformatics Group, Max Planck Institute for Molecular Plant Physiology, Am Mühlenberg 1, 14424 Potsdam-Golm, Germany

## Abstract

**Background:**

The study of biological interaction networks is a central theme of systems biology. Here, we investigate the relationships between two distinct types of interaction networks: the metabolic pathway map and the protein-protein interaction network (PIN). It has long been established that successive enzymatic steps are often catalyzed by physically interacting proteins forming permanent or transient multi-enzymes complexes. Inspecting high-throughput PIN data, it was shown recently that, indeed, enzymes involved in successive reactions are generally more likely to interact than other protein pairs. In our study, we expanded this line of research to include comparisons of the underlying respective network topologies as well as to investigate whether the spatial organization of enzyme interactions correlates with metabolic efficiency.

**Results:**

Analyzing yeast data, we detected long-range correlations between shortest paths between proteins in both network types suggesting a mutual correspondence of both network architectures. We discovered that the organizing principles of physical interactions between metabolic enzymes differ from the general PIN of all proteins. While physical interactions between proteins are generally dissortative, enzyme interactions were observed to be assortative. Thus, enzymes frequently interact with other enzymes of similar rather than different degree. Enzymes carrying high flux loads are more likely to physically interact than enzymes with lower metabolic throughput. In particular, enzymes associated with catabolic pathways as well as enzymes involved in the biosynthesis of complex molecules were found to exhibit high degrees of physical clustering. Single proteins were identified that connect major components of the cellular metabolism and may thus be essential for the structural integrity of several biosynthetic systems.

**Conclusion:**

Our results reveal topological equivalences between the protein interaction network and the metabolic pathway network. Evolved protein interactions may contribute significantly towards increasing the efficiency of metabolic processes by permitting higher metabolic fluxes. Thus, our results shed further light on the unifying principles shaping the evolution of both the functional (metabolic) as well as the physical interaction network.

## Background

To ensure stable and efficient of metabolic processes in cells, highly coordinated molecular interactions of the involved enzymes and metabolites are necessary. The study of spatially organizing principles of metabolic pathways has long been a research focus of cellular and molecular biology. Organelle compartmentalization and the organization of enzymatic pathways in so-called metabolons have been discussed as the main cellular-scale as well as molecular-scale organizational units to orchestrate the multiple metabolic processes inside cells and to separate as well as to integrate them in space and time. First introduced by Srere, the term metabolon describes a non-covalent association of several sequential enzymes involved in a metabolic pathway [1]. Similar to industrial assembly lines, intermediates are passed on from one enzyme to the next, referred to as metabolic channeling, leading to an optimized metabolic flux. The stability and structural integrity of metabolons varies greatly ranging from temporary associations and their dynamic formation in response to environmental changes to stable, permanent enzyme complexes [2,3]. Furthermore, it was found that enzyme complexes are often associated with intra-cellular membrane systems [4-6] demonstrating that the spatial organization of the metabolic network is not only limited to direct physical interaction of participating enzymes, but that it also involves passive – in the context of enzymatic pathways – mediating structural cellular components.

Metabolic channeling provides several advantages such as an increase of catalytic efficiency by shorter transition times between the consecutive active sites [7,8], local enrichment of substrates, protection from toxic intermediates by shielding them from the cellular environment, prevention of decomposition of unstable chemical compounds [9], overcoming of thermodynamically unfavorable equilibria [10-12], as well as avoidance of competitive pathways [1,13-17]. Although the concept of metabolic channeling has been discussed controversially at times [5], it is now supported by metabolic control analysis as well as experimental evidence [5,18-23].

Recently, Huthmacher and co-workers analyzed the metabolic networks of yeast and *Escherichia coli *in the context of direct protein interactions as observed in newly available, large-scale protein-protein interaction surveys allowing a systematic scan for direct protein interactions of consecutive metabolic pathway enzymes [24,25]. They found higher frequencies of physical interactions of enzymes sharing at least one common metabolite in the network. The chance for enzymes to physically interact was observed to be negatively correlated to the distance between enzymes in metabolic network in *E. coli *and, to a lesser degree, in yeast as well. In addition, they reported a higher probability of regulating enzymes to interact with other proteins, where regulating enzymes were defined either by a threshold of Gibbs free energy change of the associated reaction or by their position within the network as being located at highly connecting branching points. Furthermore, the analysis of high-throughput protein-protein interaction data yielded a number of novel candidates for metabolic channeling. Thus, the functional significance of protein-protein interactions for the metabolic pathway organization has been established and is supported by many experimental observations.

Here, we aim to expand the view on protein interactions in the context of metabolic pathways by treating both levels of molecular organization as network graphs and to investigate global as well as local network properties. The representation of complex biological networks as graphs and the study of their properties have contributed to an emerging system-wide approach towards studying the organizing principles of cellular and molecular processes. Global topological graph properties such as the degree distribution have received particular attention and have been discussed in the context of network stability and information exchange within networks [26-31].

The integrated analysis of different network types for different levels or domains of molecular organization has been applied to transfer evidence to support particular interactions from one network type to another. Ge et al. showed that gene expression and protein interaction data are correlated [32]. Kemmeren and co-workers as well as Deane et al. used gene expression data to assess confidence levels for protein interaction networks [33,34]. Goldberg and Roth predicted genetic interactions by utilizing protein interaction data [35], and Kelly and Ideker to predict the physical context of genes [36]. Rhodes et al. used GO-annotations, integrated interlogs and expression data as well as data of protein domains, known to interact to predict protein interactions [37]. The use of gene co-expression data to identify protein interactions has also been demonstrated recently [38]. Finally, Lee et al. integrated expression, gene-fusion, phylogenetic profile, literature co-citation as well as protein interaction data to predict functional associations [39].

In this study, we expand on the study of Huthmacher and co-workers by investigating the entire protein interaction network and its significance for metabolic networks and metabolic pathways. We extended enzymatic physical interactions to also include non-enzymatic proteins as metabolic relationships between enzymes may also be mediated by metabolically inactive interface proteins. Specifically, we investigate whether large-scale topological equivalences of both the metabolomic and protein interaction network can be detected. Furthermore, as the physical organization of metabolic pathways is likely to have been under evolutionary optimization to increase metabolic throughput, we are studying here whether available flux data can be correlated to the protein interaction data supporting this hypothesis. So far, protein interaction data have been analyzed primarily across all functional categories. Here, we compare the general organization principles with those observed for the enzymatic protein subset, and report that, indeed, specific differences do exist. The significance of topological parameter distributions have largely been analyzed within the context of the examined network type itself, but not across different network types. For example, Macdonald and co-workers discovered defined relationship between fluxes going through metabolic network edges and the degree product of the connected nodes [40]. Here, we explore whether such relationships can be established across network types, in particular protein interaction and metabolic networks.

Thus, our investigations aim to establish whether unifying principles shaping the evolution of both protein interactions as well as metabolic pathways can be detected.

## Results

### Topological Properties of Interaction Networks

We start our investigations by first characterizing the global network properties of the various types of molecular networks examined in this study. Besides the two main network types, the protein interaction network and the metabolic network, further filtering and different construction methodologies were applied to reveal organizational differences between raw networks including all interactions, and networks designed specifically to capture aspects of metabolism and to also safe-guard against possible artifacts resulting from a particular reconstruction scheme.

#### Protein Interaction Networks (PIN)

The raw PIN (rPIN, see Methods) derived from the merged databases of DIP and BIOgrid comprises 5,438 proteins involved in 39,766 physical interactions. The network does not differentiate whether the interaction between two proteins is transient or permanent, or under which conditions the proteins were found to interact, or the functional relevance of the association. As the PINs used in this study are represented as undirected graphs, the functionality of an interaction cannot be resolved. A kinase interacting with a protease may activate the protease or be degraded by it.

**Figure 1:**
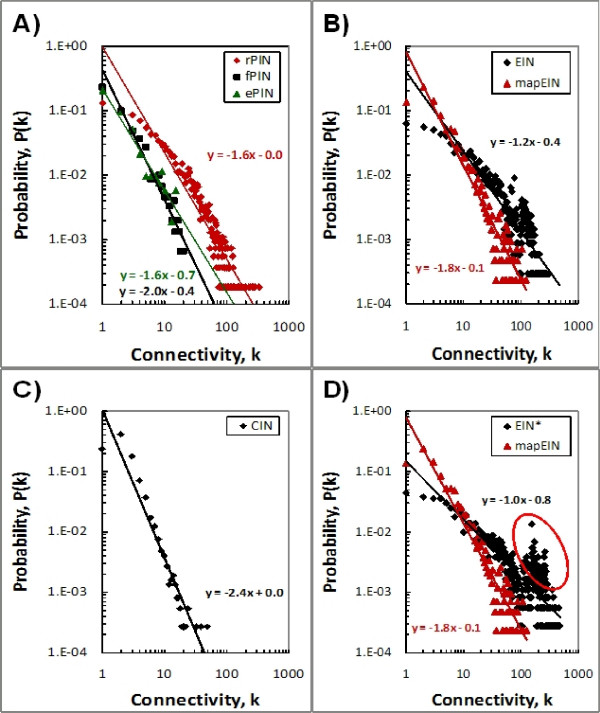
**Connectivity distribution *P(k) *for the raw (rPIN), filtered (fPIN) and enzymes-only ePIN (A) as well as EIN, EIN from KEGG-Maps, mapEIN (B) and CIN (C)**. All distributions show approximately power law behavior *P(k) *≈ *k*^-γ^, where *k *is the degree of a node, and the slope γ representing the scale-free exponent given in the graphs with some noteworthy deviations for the rPIN and the EIN. The EIN*, a variant of EIN that includes interactions derived from relations (D) between small ubiquitously occurring molecules such as H^+^, NH_3_, H_2_O, CO_2 _and metals as well as co-enzymes and co-substrates, such as CoA, NADH+, FAD, SAM (see Additional File 4), is characterized by a distribution deviating from the power law distribution (red oval) for high connectivity values, *k*. The solid lines correspond to linear regression lines applied to the raw data in log-log scale with the associated linear equation indicated in the graph.

The connectivity distribution *P(k) *of the rPIN can be approximated by a power-law function with *P(k) *≈ *k*^-γ^, where γ – the scale-free exponent – is the slope of the linear regression line in the double-logarithmic diagrams (Figure 1A). The value of γ was observed as 1.6 for the rPIN. The deviation from a straight line in the double-logarithmic suggests that a better fit may be obtained by introducing a mixture of power law and exponential degree distribution as was observed similarly for the Drosophila protein interactome [41] and other molecular networks [42-45]. As is typical for biological networks, the great majority of proteins show a small number of links whereas few proteins have up to 330 interactions. The rPIN network graph is characterized by a relatively short characteristic length (*CL*) of 3.49 ± 0.01. Of the 39,766 physical interactions, 15,232 occurred in the cytosol, 58 between two membrane associated proteins, and 298 were interactions between a membrane associated protein and a soluble protein. The sub-cellular localization information of 16,739 interactions was incomplete. GO-cellular component annotations for 7,439 interactions were inconsistent; i.e. participating proteins were reported in different compartments, and have thus been discarded from the analysis.

To analyze aspects of the protein interaction network that are specifically associated with metabolic functions, we identified proteins of rather non-metabolic functions and processes and their associated interactions. The rPIN comprises 1,186 proteins related to DNA processing functions with 21,952 associated interactions, 297 protein-degradation related proteins involved in 4,999 interactions, and 267 kinase-phosphatase associated proteins with 8,251 associations as well as 2,300 other-non-metabolic rather unspecific proteins involved in 34,230 interactions. All these interactions were partially overlapping as proteins form different groups were also reported to interact. After removing these interactions, the remaining nodes span a graph of 1,517 proteins, which can be considered to be the key molecular components responsible for maintaining the metabolic machinery. We will refer to this graph as the *filtered *PIN (fPIN). Of the 1,517 proteins, 522 represent enzymes annotated with an EC-number. The fPIN comprises 1,086 links, with 289 interactions between enzyme pairs. One third of all nodes are included in the graphs giant component, the largest connected sub-graph. We left unconnected nodes in the graph as the absence of interactions of such proteins may also be significant. In comparison to the raw network, the number of enzymes (869 in the rPIN) is lower, because in the fPIN, non-metabolic enzymes such as protein kinases and protein phosphatases have been excluded.

Further removal of proteins not assigned to at least one EC-number led to the enzyme-only-PIN (ePIN), a graph comprising only enzymes and the interactions between them. Its giant component contains 19% of all nodes. Thus, with applied filtering, the PIN became progressively disintegrated.

As observed for the rPIN, and even more convincingly, the connectivity distribution of both networks, the fPIN and ePIN, follows a power law behavior with respective scale-free exponents of 2.0 for the fPIN, and 1.6 for the ePIN (Figure 1A). Compared to the rPIN and explained by the removal of many non-specific interactions, the fraction of highly-connected nodes is reduced in the fPIN and ePIN with a simultaneous increase of unconnected nodes. The characteristic length (*CL*) of the fPIN is 8.16 and for the ePIN 6.22, which is approximately twice as long as the *CL *associated with rPIN (3.49) suggesting that, in particular, highly connected nodes providing shortcuts have been removed in the fPIN and ePIN compared to the rPIN even though the networks as such are smaller as nodes have been deleted. Note that impossible paths (no connection between nodes) have not been included in the calculation of *CL*.

**Figure 2:**
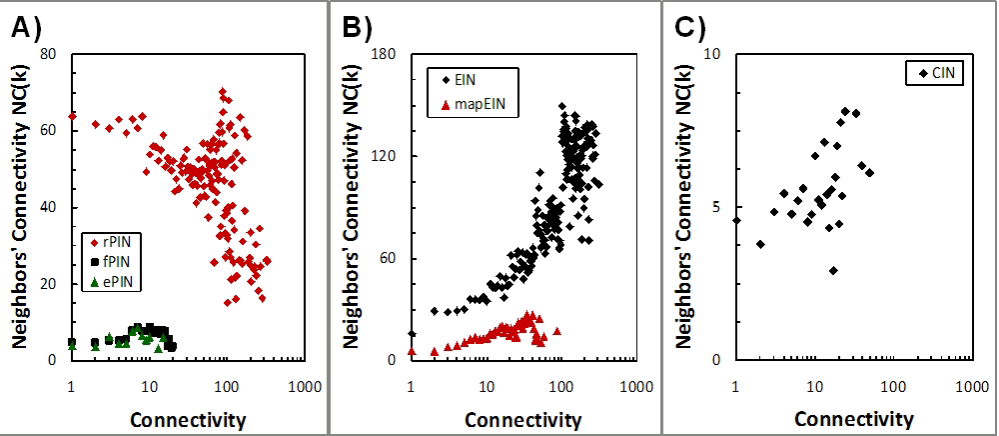
**Neighbors' Connectivity (NC) as a function of connectivity of the respective reference node for the (A) rPIN, fPIN and only ePIN as well as EIN, (B) mapEIN, and (C) CIN**. Plotted points correspond to mean values at a particular connectivity value. Linear regression analysis applied to the raw NC-connectivity value pairs yielded for the rPIN (Pearson correlation coefficient, *r*, and associated p-value, *p*): *r *= -0.10, *p *= 9.7E-15; fPIN: *r *= 0.16, *p *= 7.1E-6; ePIN: *r *= 0.21, *p *= 1.3E-3; EIN: *r *= 0.70, *p *= 0; mapEIN: *r *= 0.47, *p *= 2.6E-109; and CIN: *r *= 0.08, *p *= 1.9E-7. Note: the number of raw values per plotted mean value decreases rapidly with increasing connectivity. Thus, the apparent cluster of points in the rPIN near connectivity values near 100 is not statistically significant.

The average cluster coefficient of the rPIN was determined as 0.16, 0.39 for the fPIN, and 0.41 for the ePIN indicating increased modularity of the two filtered PINs compared to the raw protein interaction network. While the rPIN shows a negative correlation of degrees associated with neighboring nodes, i.e. it is dissortative, the fPIN and the ePIN revealed a positive correlation. The assortativity (*r*_*d*_) was calculated as -0.11 for the rPIN, 0.15 for the fPIN, and 0.16 for the ePIN. All correlations are significant with associated p-values of 1.0E-101, 1.0E-6, and 0.005, respectively. The negative correlations in the rPIN can be explained by the high dissortativity of protein sub-networks that have been discarded in the filtered PINs. The graph comprising relations between kinase-phosphatase associated proteins shows a dissortativity of -0.36, DNA-related proteins of -0.12, protein-degradation -0.26 and other-non-metabolic proteins of -0.22. Consistent with these findings, the distribution of the neighbors' connectivity increases with increasing connectivity of nodes for the fPIN and ePIN, albeit moderately – yet significantly, and decreases for the rPIN with increasing degree of nodes (Figure 2A).

Thus, the organizing principles governing protein interactions between proteins involved in metabolic functions appear to be different than for other functional categories. While PINs generally are dissortative, protein interactions associated with metabolic functions appear to be assortative; i.e. enzymes preferentially interact with other enzymes of similar degree (connectivity).

#### The Metabolic Interaction Networks (MIN)

We analyzed three different realizations of metabolic interaction networks (MIN) each representing metabolic pathways from a different perspective. The first two representations of a metabolic interaction networks are the Enzyme Interaction Networks (EIN) and the EIN derived from KEGG pathway maps (mapEIN), where the nodes of the graph are enzymes with assigned EC-numbers. In the EIN, two enzymes are linked if they are associated by at least one product-substrate relationship. For constructing the mapEIN, we extracted relations from KEGG pathway maps directly rather than scanning reaction lists for product-substrate relationships as done for the EIN. While the EIN comprises a large number of enzymes and their relations, the mapEIN may capture better the established biochemical knowledge of metabolic pathways. The Compound (Metabolite) Interaction Network (CIN) represents a third representation of MINs. In this graph, nodes are metabolites, and links are drawn between them if they are connected by at least one reaction.

The EIN comprises 3,435 nodes representing unique EC-numbers. The connectivity distributions, *P(k)*, of the graph follows approximately a scale-free distribution with an estimated scale-free exponent γ of 1.8 (Figure 1B). As observed for the rPIN, a deviation from a simple power law behavior is evident (see above). However, the distribution follows a power law only if small ubiquitously occurring, so-called currency metabolites, such as H^+^, NH_3_, H_2_O, CO_2_, and metal ions as well as co-enzymes and co-substrates, like CoA, NADH+, FAD, SAM are excluded (Figure 1D). Including these compounds significantly increases the degree of the enzymes interacting with them resulting in a distribution *P(k) *deviating from the power law distribution for high connectivity values (Figure 1D). Upon including currency metabolites, the total number of edges increases from 60,622 to 140,260 and characteristic length, *CL*, decreases from 3.64 to 3.00.

The mapEIN comprises 1,957 nodes connected by 6,395 relations. The scale-free exponent of the connectivity distribution, γ, was computed as 1.2 with an increased probability of nodes to be less connected as compared to the EIN, where many more relationships between enzymes are possible simply via their possible substrate-product relationships. The *CL *of the mapEIN network was determined as 6.62.

The third representation, the CIN, comprises 3,702 metabolites connected by 4,868 links. As done for the EIN, the currency metabolites, co-enzymes and co-substrates have been removed prior to analysis. The connectivity distribution of the CIN exhibits a scale-free exponent, γ, of 2.4 and CL of 12.3 (Figure 1C).

All three MIN graphs are assortative with assortativity values, *r*_*d*_, of 0.43, 0.26, and 0.09 in the EIN, mapEIN, and CIN, respectively. Correspondingly, an increasing neighbors connectivity, *NC(k)*, was observed for increasing connectivity, *k *(Figure 2B, C). The high assortativity value for the EIN probably results from the construction procedure. The EIN was constructed by scanning for product-substrate relationships. As reactions are generally treated as reversible, so that the lists of substrate and products are interchangeable, all enzymes sharing a metabolite may be linked through substrate-product relations and form a complete sub-graph. While the high assortativity of the EIN may originate from the reconstruction method possibly resulting in too many connections, this may not be the case for the mapEIN as the interactions have been curated manually. However, many reactions in the KEGG-maps are known to be performed by isoenzymes carrying different EC numbers. Since reactions are treated as reversible, isoenzymes will be considered connected as the product of one isoenzyme can be the substrate of another, even though it is the same reaction they are catalyzing. Thus, a set of isoenzymes will form a fully-connected sub-graph, also including the enzymes of the preceding or subsequent reaction step as each isoenzyme is connected to them. The reconstruction of the CIN avoids this problem. This third representation of MINs is closest to the biological and intuitive understanding of metabolic pathways. A pathway in this sense is the path from a first substrate to a final product. The difference in the respective construction methods is also reflected by the average clustering coefficient (CC), where the CC for EIN was 0.67, EIN from KEGG-Maps 0.47, and 0.06 for the CIN, respectively.

A summary of global network properties for the PINs and MINs investigated in this study is provided in Table 1.

**Table 1:** Summary of global network properties associated with the different types of PINs and MINs investigated in this study.

	PIN					
	rPIN	fPIN	ePIN	EIN	mapEIN	CIN
Number of nodes	5438	1517	522	3435	1957	3702
Number of edges	39766	1086	298	60622	6395	4868
Number enzymes	869	522	522	3435	1957	n.a.
Giant component	5415	510	99	3276	1674	3374
Characteristic length, CL	3.64	8.16	6.22	3.64	6.62	12.30
<Cluster coefficient>	0.16	0.39	0.41	0.67	0.46	0.06
<Neighbors' connectivity>	57.17	2.65	1.97	55.60	10.34	4.41
Assortativity	-0.11	0.15	0.16	0.43	0.26	0.09

Brackets indicate mean values. Properties are explained in greater detail in the Methods section.

#### Correlation of protein interaction networks (PINs) and associated metabolic interaction networks (MINs)

Nodes in the EIN and mapEIN represent enzymes. It is therefore possible to link enzymes found in PINs to the EIN and mapEIN via their annotated EC-numbers. Enzymes from PINs can be linked to metabolites from the CIN network via the enzyme (EC-number)-substrates and -product relationships. Thus, it is possible to directly relate network distances of proteins (enzymes) across both network types (PINs and MINs) allowing us to study, how metabolic network or pathway distances are reflected in protein interaction networks.

**Figure 3:**
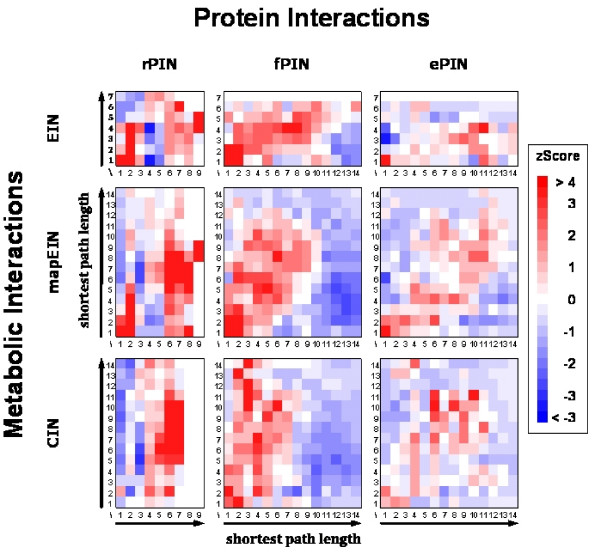
**Enrichment and depletion of the abundance of shortest path pairs between nodes represented in both the PIN and MIN**. The enrichments and depletions were judged by the *z*-score of the frequency of observations in comparison to randomized distribution with red-color indicating enrichment and blue-color depletion relative to randomized networks.

We evaluated the distribution of the shortest paths between distance pairs in the PINs and MINs, comparing the actually observed distribution to distributions generated by 1,000 randomly constructed networks (see Methods). The over- or under-representation of the distances were judged by the z-score of observed frequencies (Figure 3, raw frequency counts are available in the Additional File 1). We applied the analysis to all PINs and related them to the EIN, the mapEIN, and the CIN.

A direct correspondence between the protein interaction networks and metabolic networks; i.e. the physical organization of enzyme interactions follows directly their reaction pathway network, would be reflected by red-colored squares – indicating increased occurrence compared to random expectation – along the diagonal in the pair-distance matrices shown in Figure 3. Indeed, the distributions of the enrichments and depletions of the distance-pairs reveal an overall correlation of the shortest paths in PINs and MINs. All PINs show a strong enrichment of direct interactions; i.e. distance 1, in relation to the EIN and mapEIN. Furthermore, an overall correlation of distance pairs with increased numbers of observations relative to the random background (red squares along the diagonal, blue squares primarily off-diagonally) for all PIN-MIN comparisons is evident, especially for the fPIN (Figure 3, central column). Interestingly, enzymes appear more closely related (shorter distances between them) in the fPIN in comparison to their distances derived from their metabolic network association (mapEIN and CIN), as the off-diagonal pattern of red-colored squares indicates a skewed distribution towards larger shortest paths between linked proteins in the MIN compared to their distances in PINs. Thus, it appears that enzymes catalyzing enzymatic steps of some medium distance, i.e. not directly subsequent to each other, are physically brought into contact via protein-protein interactions involving proteins that are not necessarily directly participating in the actual metabolic pathway. For the EIN, above-diagonal enrichments were not observed when compared to the fPIN and ePIN, possibly a consequence of the network reconstruction procedure that allows very many interactions leading to a highly connected metabolic network. As the fPIN contains both enzymes and structural proteins, some proteins included in this network may function as connector or bridging proteins holding distant parts of metabolic pathways together. In the ePIN, such proteins have been filtered out leaving only enzymes in the PIN. Here, the enrichment pattern follows the main diagonal, but at weaker significance as the absolute numbers are smaller. A direct comparison of shortest paths between enzyme pairs connected via valid paths in both the fPIN and the ePIN yielded a mean distances of 5.3 for the fPIN, and 6.2 for the ePIN (p = 0; paired, two-tailed t-test, N = 5,531). Thus, metabolic enzymes are brought into spatial proximity – by way of protein-protein interactions – via interactions mediated by non-metabolically active proteins.

The overall Pearson correlation values, *r*, for distance-pairs (PIN, MIN) are listed in Table 2. All correlations are highly significant (p < 1.0E-40). Thus, in all comparisons, a positive correlation of the organization of protein-protein relations was observed between their enzymatic pathway organization and their corresponding physical organization. The correlation is strongest when enzyme-only protein networks are compared to MINs, in particular to KEGG-map derived metabolic pathways (mapEIN). The PIN-MIN correlations were observed to become more pronounced when more relevant (with regard to metabolism) PINs were considered and increase steadily from rPIN to fPIN, with greatest correlations observed for the ePIN. Thus, it is no contradiction that the correlations for the rPIN are low, but a result, because many unspecific interactions included in the rPIN were eliminated in the other PINs. The reported correlation coefficients (Table 2) were computed over the entire range of network distances including distant pairs for which correlations can be expected to be low. Correspondingly, correlation values increased significantly when remote pairs were discarded (Additional File 2).

**Table 2:** Correlation between pairwise network distances of proteins in MINs compared to their respective distance in PINs.

		**PIN**		
		**rPIN**	**fPIN**	**ePIN**
**MIN**	**EIN**	0.07	0.18	0.35
	**mapEIN**	0.09	0.21	0.44
	**CIN**	0.09	0.14	0.22

Listed are the Pearson correlation coefficients, *r*. All correlations were highly significant (*p *< 1.0E-40).

#### The correlation of metabolic fluxes carried by enzymes and their Protein Interaction Network properties

**Figure 4:**
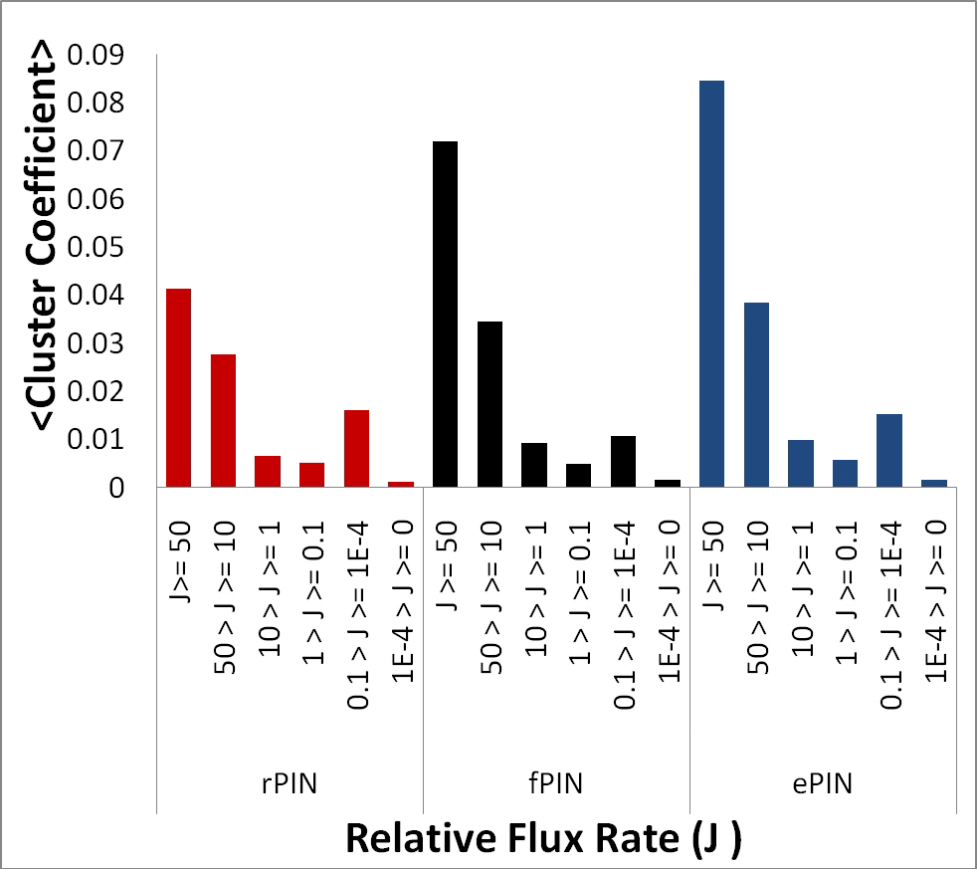
Average PIN clustering coefficient of enzymes binned by relative flux rates, *J*.

On the basis of measured relative metabolite flux rates of yeast growing in a glucose medium, we evaluated the correlation of network cluster coefficients of the involved enzymes in PINs to the flux rate carried by the enzymes. The flux rates were estimated by Blank and colleagues based on a global metabolic network model of *S. cerevisae *[46] and a flux balance analysis based on large-scale ^13^C-isotope tracer experiments [47]. Our analysis revealed high PIN clustering coefficients for high flux enzymes decreasing with the decrease of the relative flux rates (Figure 4). Further analysis revealed that the connectivity as well as the betweenness centrality are also positively correlated with the flux rates carried by the associated enzymes (Table 3). Thus, highly connected and central enzymes (in PINs) are enzymes carrying high fluxes. Furthermore, enzymes preferentially interact with enzymes of similar flux rates. A strong positive correlation of flux rates of physically interacting proteins was observed (correlation coefficient of 0.52) in the fPIN and ePIN.

**Table 3:** Correlations between the relative flux rates between neighboring (physically interacting) enzymes and their PIN properties

	**rPIN**		**fPIN**		**ePIN**	
**Correlation between**	**r**	**p-Value**	**r**	**p-Value**	**r**	**p-Value**
Connectivity~Flux rate of nodes	0.15	4.76E-06	0.23	1.08E-04	0.25	8.99E-05
Centrality~Flux rate of nodes	0.14	2.16E-05	0.11	1.24E-02	0.32	9.34E-13
Flux Rate~Flux rate of neighbors	0.24*	5.25E-25	0.52	1.20E-41	0.52	1.45E-40

Pearson correlation coefficient, *r*, and associated *p*-values between connectivity (degree), centrality as judged by betweenness of enzymes (nodes in PINs) and the respective relative flux rates, as well as the correlation coefficient of the relative flux rates between neighboring (physically interacting) enzymes in the three PINs examined in this study.

*Correlation coefficient, *r*, differs in rPIN from values obtained in fPIN and ePIN, because enzyme pairs are included in the rPIN that were filtered out in the other two PINs because of inconsistent subcellular localization annotation. They are identical for the fPIN and ePIN as the two networks contain the same set of enzymes.

#### Physical interactions in high-throughput catabolic pathways and synthesis pathways of complex metabolites

**Figure 5:**
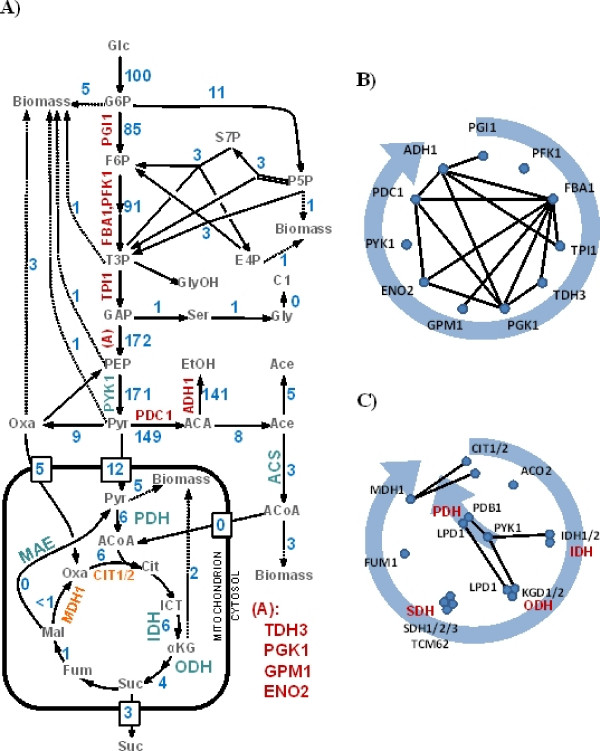
**(A) Generalized view of the utilization of glucose by yeast growing in a glucose medium**. Only reactions with relative flux-rates greater than 1 relative to a glucose uptake of 100 (arbitrary flux units) are shown (blue numbers). The protein names represent enzymes responsible for the respective reaction. Three notable interaction clusters of enzymes can be found indicated by orange, cyan, and red color. (B) Interaction cluster of enzymes involved in fermentative glycolysis. The enzymes are ordered in clockwise direction according to the catabolic step during the process. Black edges indicate interaction in the protein-protein interaction network. (C) Interaction clusters of enzymes of the TCA-cycle as well as the prior reactions of the pyruvate kinase (PYK1) and pyruvate dehydrogenase (PDH).

To gain further insight, we studied the physical organization of enzymes carrying high fluxes in greater detail. The large-scale flux analysis in yeast by Blank and co-workers [47] comprised 1,038 reactions (745 distinct reactions) encoded by 672 genes of which 610 can be found in the rPIN. Of the distinct reactions, 28% (208 reactions) have reaction rates greater than 1 relative to a glucose uptake of 100 (arbitrary flux units) and can be summarized in a global glucose utilization scheme (Figure 5A). In this scheme, 16 reactions, 2.1% of the 1,038 reactions considered by Blank, have flux rates greater than 50, corresponding to 61 proteins found in the PIN and were contained in all three PIN variants studied here. The reactions include two transport reactions of the products of the fermentative glycolysis with no annotated gene assigned to these steps, the glucose uptake, and the reaction performed by the ATPase complex. The remaining reactions are involved in the fermentative glycolysis as shown in Figure 5B. Glycolysis describes the utilization of glucose as an energy source upon its degradation to pyruvate. Depending on the culture conditions, pyruvate may either be fully degraded to CO_2 _by the enzymes of the TCA-cycle within the aerobic glycolysis, where the pyruvate dehydrogenase (PDH) connects glycolysis with the TCA-cycle enzymes, or to ethanol by pyruvate decarboxylase (PDC) and alcohol dehydrogenase (ADH) within the fermentative glycolysis, when O_2 _is limiting. The enzymes of the fermentative glycolysis are highly interconnected with each other with many physical interactions detected between the associated enzymes (Figure 5B). An exception is the pyruvate kinase (PYK1, CDC19) which is not physically linked to any of the other enzymes of the pathway, as well as the 6-phosphofructokinase (PFK). Instead, PYK interacts with PDH.

Only a minor fraction of pyruvate (flux rate of 6 relative to the glucose uptake rate flux rate set to 100) appears to be channeled to the TCA-cycle, that is 3% (as one glucose molecules may lead to the formation of two pyruvate molecules) of the initial glucose influx is processed by the TCA-cycle enzymes. The production of pre-stage substrates of amino acids rather than energy production is the main function of the TCA-cycle. The flux rates decrease to 1 beyond the succinate dehydrogenase (SDH) reaction step. This path leads through the PYK1, PDH and the following enzymes of the TCA-cycle: citrate synthase (CIT), isocitrate deyhdrogenase (IDH), 2-oxoglutarate complex (KGD), succinlyCoA synthetase (LSC) and SDH. The enzymes of the TCA exhibit a relatively low number of physical interactions (Figure 5C). The interactions are mainly pooled in enzyme complexes, SDH (SDH1/2/3, TCM62), the LSC (LSC1/2) and KGD (KGD1/2, LPD1), performing the individual reactions steps of the TCA-cycle. Only the reactions of the malate dehydrogenase (MDH1) and the CIT are physically connected. However, taking the prior reactions of the PYK1 and PDH into account, the TCA reactions reveal a more dense interaction cluster. The PDH interacts with KGD sharing the common subunit lipoamide dehydrogenase (LPD1). The PYK1 interacts with PDH, KGD as well as IDH (Figure 5C).

The remaining reported direct physical interactions contained in the fPIN between enzymes detected within metabolic pathways are distributed throughout anabolic pathways. Most interactions are found in the biosynthesis of ergosterol, ubiquinone, sphingolipid and glucogen synthesis (Additional File 3). Single links between enzymes can be found in biosynthetic pathways of pyrimidine, leucine, isoleucine, and lysine. Within the fatty acid synthesis pathway, the malic enzyme (MAE1) interacts with the alpha subunit of FAS and Acetyl-CoA carboxylase (ACC1) (Additional File 3)

Figures 5B, C and the Additional File 3 provide a comprehensive account of all reported protein interactions mapped to canonical metabolic pathways from the SGD database; i.e. for pathways not included in this figure, no protein interaction was contained in the PIN.

#### Central proteins in the fPIN

**Figure 6:**
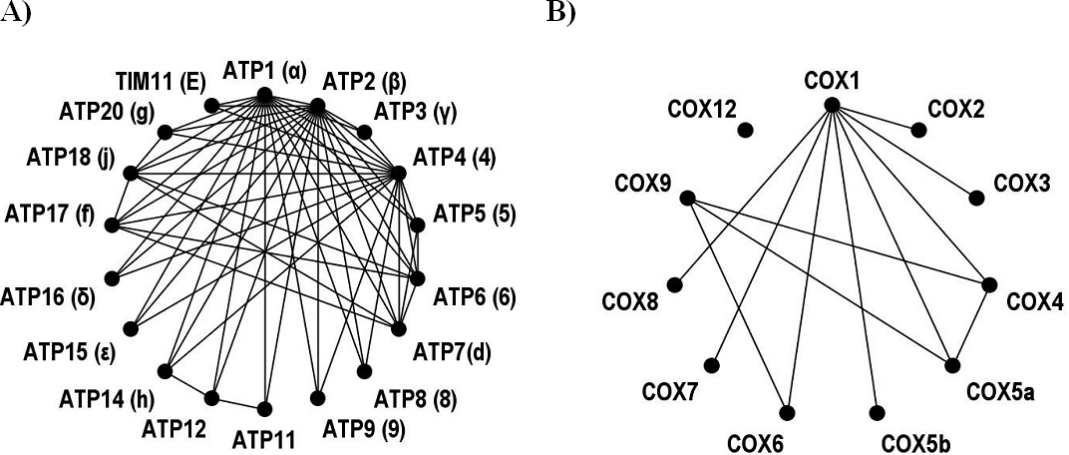
**The physical interaction clusters of the A) ATP synthase, and B) Cytochrome c**. The identity of enzymes is given by their gene symbols and detected interactions between them denoted by solid lines.

Analyzing centrality as judged by the z-score of the change of the characteristic length of the graph after removal of a particular node identified enzymes with the most influence on the cohesion of the interactome. The ten most influencing proteins are listed in Table 4. ATP14 exhibits the most influence on the characteristic length of the fPIN. The H-chain of the ATP synthase is one of 17 polypeptides building up the complex (Figure 6A). While only interacting with a relatively low number of other subunits of its own complex, it interacts with the Complex IV (Cytochrome c) of the respiratory chain, via COX5B and Complex III (Cytochrome b-c1) via QCR8. Furthermore, it interacts with the FBA1 from the glycolysis pathway, a central enzyme assembling the glycolytic cluster.

**Table 4:** Ten most central proteins in the fPIN.

**Protein**	**RB**	**BN**	**DC**	**Protein description**
ATP14	17.17	25.44 (1)	5.14 (14)	ATP synthase H chain, mitochondrial
FBA1	11.34	6.71 (6)	4.45 (20)	Fructose-bisphosphate aldolase
TSC13	8.49	10.28 (3)	3.07 (33)	Enoyl reductase, very long fatty acid elongation
IFA38	7.73	1.26 (37)	8.59 (4)	Oxidoreductase, fatty acid elongation
COX1	7.69	4.43 (14)	5.14 (15)	Cytochrome c oxidase subunit 1
SER3	7.49	-0.32 (1449)	5.83 (8)	D-3-phosphoglycerate dehydrogenase 1
COX5B	7.19	3.53 (17)	1.00 (117)	Cytochrome c oxidase polypeptide Vb, mitochondrial [Precursor]
URA2	6.74	2.72 (21)	3.07 (31)	Bifunctional glutamine-dependent carbamoyl-phosphate synthase, Aspartate carbamoyl-transferase
GPM1	6.60	2.94 (20)	2.38 (42)	Phosphoglycerate mutase 1
MAE1	6.46	9.46 (4)	0.31 (160)	NAD-dependent malic enzyme, mitochondrial [Precursor]

Ten most central proteins in the fPIN as judged by the *z*-score of the change of the characteristic length of the graph after removal of a particular protein, robustness centrality (RB). For comparison to other centrality measures, the *z*-scores and respective rank (in parentheses) of the betweenness centrality (BN) as well as degree centrality (DC) were evaluated. The overall correlation between the centrality was RB~BN 0.70 (*p*-Value: 4.35E-220), RB~DC 0.59 (*p*-Value: 1.44E-144) and BN~DC 0.25 (*p*-Value 3.37E-23)

**Figure 7:**
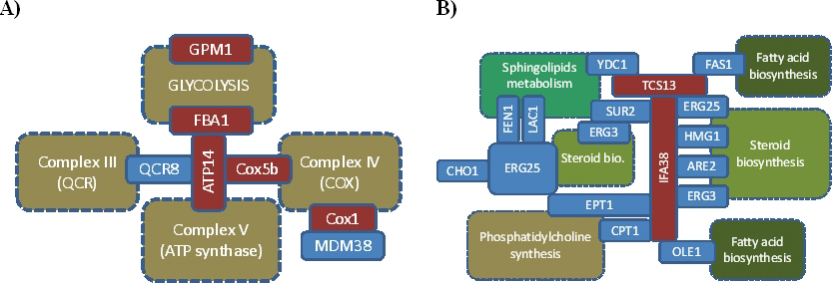
**Crosstalk via protein interactions between enzymes and proteins in A) glycolysis and the respiratory chain (ubiquinol cytochrome-c reductase complex (Complex III); cytochrome c oxidase (Complex IV) and ATP synthase (Complex V)) as well as B) systems for biosynthesis of membrane lipids (biosynthetic systems for sphingolipids, fatty acid, steroids as well as for phosphatidylcholine)**. Individual proteins are highlighted in blue. If belonging to the group of 10 most central proteins of the fPIN (see Table 4), they are colored red. Boxes with dashed perimeter indicate larger metabolic systems. Touching protein boxes indicate interaction between the proteins, whereas protein boxes emerging from metabolic system denote participation of that protein in this system. ARE2: Acyl-CoA:sterol acyltransferase; ATP14: H chain of ATP synthase; CHO1: phosphatidylserine synthase; Cox5b and Cox1: Subunit Vb and I of cytochrome c oxidase; CPT1: Cholinephosphotransferase; EPT1: sn-1,2-diacylglycerol ethanolamine- and cholinephosphotranferase; ERG25: C-4 methyl sterol oxidase; ERG3: C-5 sterol desaturase; FAS1: Beta subunit of the fatty acid synthase; FBA1: Aldolase; FEN1: Fatty acid elongase, involved in sphingolipid biosynthesis; GPM1: phosphoglycerate mutase; HMG1: HMG-CoA reductase; IFA38: oxidoreductase, fatty acids elongation; LAC1: Ceramide synthase; MDM38: required for k+/H+ exchange; OLE1: Delta(9) fatty acid desaturase; QCR8: Subunit 8 of ubiquinol cytochrome-c reductase complex; Sur2: Sphinganine C4-hydroxylase; TSC13: enoyl reductase, very long fatty acids elongation; YDC1: Alkaline dihydroceramidase, sphingollipids metabolism;

COX1 and COX5B are two of 11 subunits of Cytochrom b-c1 (Figure 6B). While COX1 plays an essential role in the assembly of the complex, the role of COX5b is the interaction with ATP synthase. The FBA1 and GPM1 are part of the glycolytic cluster. Taken together, the glycolysis pathway and the respiratory chain are tightly connected via physical interactions illustrated in Figure 7A.

TSC13 and IFA38, which are responsible for the elongation of very long fatty acids, connect enzymes involved in the biosynthesis of membrane lipids by interacting with enzymes from the biosynthesis of fatty acids, steroids and related metabolites, phosphatidyl -choline, -serine and -ethanol amine, suggesting that the pathways are brought into spatial proximity via protein-protein interactions (Figure 7B).

For comparison, other centrality measures for the top 10 most influencing proteins are listed in Table 4. While an overall correlation between the centrality measures is evident (correlation coefficients and associated *p*-values are provided in the legend of Table 4), each centrality measure identifies particular aspects of centrality and does not correspond directly to the robustness measure used here.

## Discussion

Our investigations integrated protein interaction networks with metabolic networks to study the extent to which metabolic pathways; i.e. functional processes, are pre-formed in the underlying structural interaction network, i.e. the "plumbing" of cellular components. The networks examined here were derived from different sources of information and provide different views on the metabolic as well as protein interaction systems.

We discovered that sub-systems of the entire protein-protein interaction network may follow specific organizing principles. While interactions associated with signaling and other regulatory processes (e.g. transcriptional regulation via DNA-interaction associated proteins) were found to be dissortative; i.e. proteins of high degree interact with proteins of low degree, interactions between metabolic enzymes were observed to be assortative such that enzymes frequently interact with other enzymes of similar degree (Table 1). Regulatory processes may often involve hierarchical one-to-many associations such as master regulators (e.g. kinases) and their respective individual target proteins. Physical interactions between metabolic enzymes, on the other hand, appear to generally follow a more horizontal organization with enzymes participating in larger complexes or sequential one-to-one interaction chains. Nonetheless, we identified interaction hub enzymes that are located at central positions integrating several metabolic systems and whose removal would severely impact the structural integrity of larger portions of the metabolism-focused metabolic network (fPIN, Table 4, Figure 7A, B).

When dealing with characteristics of protein-protein interaction network, possible technological as well as biases introduced by targeted scientific interest always are a concern. To best avoid this problem, it would be ideal to use strictly unbiased datasets for analysis. However, such fully unbiased datasets are not available (yet) as this would require nothing less than an identification of all true and relevant protein-protein interactions occurring inside cells. Presently, we have to resort to the best available unbiased datasets generated by high-throughput screens. As the BIOGRID data contains information about the source of information, it is possible to evaluate the assortativity of the biggest subsets in the database that were obtained from high-throughput experiment, namely Krogan et. al [48] comprising 1,669 nodes involved in 2,682 interactions, and Gavin et al. [49] with 2682 nodes involved in 8,138 interactions. Reducing the filtered PIN to the these subsets yields two sub-fPINs comprising 364 nodes involved in 411 interactions, and 757 nodes involved in 475 interactions, respectively. The reduced fPINs exhibit an assortativity of 0.31 and 0.15, respectively, confirming the results obtained for the whole fPIN. Correspondingly, for the rPIN, a reduced assortativity was obtained for both datasets with 0.15 for the Gavin set (N_nodes _= 1,669, N_interactions _= 10,992) and -0.01 (N_nodes _= 2,682 and N_interactions _= 8,138) for the Krogan set. Thus, given the available datasets, the increased positive assortativity of filtered/enzyme PINs does not appear to be resulting from a bias towards well-studied enzymes.

We generated three different versions of the metabolic interaction network (MIN). The enzyme interaction network, EIN, was introduced to capture all possible metabolic interactions between enzymes, whereas the mapEIN transformed the pathway knowledge available in KEGG into a metabolic network. The compound interaction network, CIN, was created as an alternative and focuses on main metabolites as network nodes rather than enzymes. With regard to our main research focus, the topological equivalence of protein interactions and metabolic pathways, all three versions yielded significant positive correlations between the respective shortest paths across both network types (Table 2). Thus, the reported results proved robust against details of the network reconstruction approach. All three MIN-versions were reported here with positive assortativity (Table 1) while a negative assortativity of metabolic networks has been reported elsewhere (degree correlation coefficient of -0.24 [50]). We note that this difference is caused by the elimination of ubiquitous (currency) metabolites and the inclusion of only main metabolic substrates and products in this work. Including all metabolites in the CIN yielded a degree correlation coefficient, *r*_*d*_, of -0.3 and an increased mean cluster coefficient of 0.7. As currency metabolites such as ATP follow a one-to-many network motif, thereby also introducing many more edges in the network, the dissortativity obtained when including them as well as the increased mean cluster coefficient can be rationalized.

The decision on the exact procedure to generate metabolic networks must remain operational and may dependent on the objective of the study at hand. Defining metabolic networks based on carbon atomic traces in metabolic reactions resulted in different topological characteristics of metabolic networks than for the commonly used approaches [45].

The interaction networks investigated in this study vary regarding their graph-parameters, such as characteristic length (*CL*) values and scale-free exponents and also differ from some networks reconstructed. Generally, biological networks tend to be scale-free with associated scale-free exponents reported below two, which was suggested to result form evolutionary mechanism driven by gene duplication [51]. However, larger exponents have been reported for the CIN [29]. Joang et al. analyzed compound interaction networks of 43 organisms. The average *CL *was observed as 3.29 ± 0.11 and the average scale-free exponent as 2.18 ± 0.09. While the scale-free exponent reported here (2.4) is in line with the reported average value, the CL reported here is much larger (12.3). The reasons for the difference can be attributed mainly to the different approaches taken to reconstruct the CIN, and the filtering mechanisms applied to remove compounds that are not directly relevant for main biochemical pathway routes such as co-factors. Here, we followed the concept of main metabolite relations introduced by Kotera and co-workers and annotated accordingly in the KEGG database [52,53]. By contrast, the networks of Joang et al. comprised all relations between all substrates and products, including currency metabolites such as H_2_O or ATP rendering the *CL *much smaller.

In their analysis of the E. coli metabolic pathway network, Wagner and Fell reported a mean *CL *of 3.8. This value compares favorably with our value (3.64) for the yeast EIN, which corresponds to the network analyzed by Wagner and Fell [30]. A similar value has also been reported by Huthmacher et al. (*CL *= 3) [25]. Similarly, the scale-free exponents agree well (-1.3 Wagner and Fell; -1.2 reported here for yeast). Kotera et al. [53] reported a *CL *of 9 for the equivalent of our CIN. The larger value we obtained (*CL *= 12.3) is explained by the exclusion of currency metabolites in our analysis. The newly introduced mapEIN (CL = 6.62) is not directly comparable to previous studies. It was constructed to capture our accumulated knowledge of biochemical pathways represented in KEGG and with nodes represented by enzymes, not compounds.

For the rPIN, our reported values for graph properties such as *CL *and scale-free exponent agree well with previously reported data [31,54]. For the other PIN types studied by us, no comparative data are available.

In their analysis of protein interaction data in the context of metabolic pathways, Huthmacher and co-workers [25] focused on direct interactions between enzymes catalyzing consecutive metabolic reaction steps. Here, we expanded the scope of an integrative analysis by also showing that such correlation between metabolic and protein interaction data is discernable even at larger distances. Of course, an increased probability for consecutive enzymes to interact naturally leads to correlations at larger distances as well, even though the significance can be expected to drop. We showed that such large-scale topological correspondence between both the PIN and MIN indeed exists adding further evidence for the significance of physical interactions for the functioning of metabolic reactions. Our analysis also revealed that shortest paths between two enzymes appear to be shorter in the PIN compared to their distance when analyzed in the metabolic network (Figure 3, elevated z-scores above the diagonal), especially when the allowed physical interactions also include proteins not actively participating in enzymatic reactions (fPIN). A direct comparison of shortest paths between enzyme pairs connected via valid paths in both the fPIN and the ePIN yielded a mean distances of 5.3 for the fPIN, where non-metabolically active proteins are still included, compared to 6.2 for the ePIN. Thus, our analyses suggest that such metabolically passive proteins may function as interface components to spatially organize enzymatic pathways.

The functional significance of topological parameters of molecular networks has largely been analyzed within the context of the examined network type itself such as the reported relationship between fluxes passing through metabolic network edges (reactions) and the degree product of the connected nodes [40], but not across different network types. Here, we showed that such relationships can also be established across different network types such that topological parameters of enzymes within the context of protein interactions have relevance for their functional, metabolic context. In particular, we observed that metabolic flux rates are positively correlated with degree and centrality of enzymes in their PIN (Table 3). We interpret this observation as evidence for a co-evolutionary adaptation of both network types. High-flux enzymes are physically interacting with many other enzymes such that metabolic substrates and products can be passed on to subsequent enzymes quickly and efficiently.

On the technical side, it has to be borne in mind that our knowledge of protein interactions is certainly incomplete and may contain many false positive interactions [55,56] and the employed technologies may skew the datasets towards particular interactions [57]. Furthermore, since we used sub-cellular localization information to eliminate potential false positive protein associations, this information, too, is to some degree based on predictions alone and may contain erroneous assignments. However, the fact that we did observe significant correlations between protein interactions and metabolic pathways despite the noise in the data may suggest that the true topological correspondence may actually be even stronger than reported here.

## Conclusion

Our results reveal topological equivalences between the protein interaction network and the metabolic pathway network. Evolved protein interactions may contribute significantly towards rendering metabolic processes more efficient by permitting increased metabolic fluxes. Thus, our results shed further light on the unifying principles shaping the evolution of both the functional (metabolic) as well as the physical interaction network.

## Methods

Because yeast represents a model organism with comprehensive experimental as well as annotation data available for both protein-protein interactions as well as metabolic reaction pathways, we focus our investigations on *Saccharomyces cerevisiae*.

### Protein Interaction Networks (PIN)

To study protein interaction networks (PINs) from a global perspective as well as by focusing on enzymatic proteins alone, we generated three different network graphs describing protein-protein interactions. The raw, unfiltered PIN (rPIN) was constructed by extracting physical interactions reported in the protein interaction databases DIP, version 20060402 [58] and BIOGrid, version 2.0.21 [59], respectively. Based on available gene ontology (GO) annotation information, proteins involved in processes related to protein translation, DNA-transcription and associated regulatory processes, such as transcription or translation factors, as well as proteins involved in the assembly of chromatin structures were labeled as 'DNA-related'. Proteins involved in degradation and related regulatory proteins were labeled as 'degradation-related', protein kinases and phosphatases and related regulators labeled as 'kinase-phophatase-related'. Additionally, we defined a set of proteins as 'other non-metabolic proteins'. This set comprised proteins assigned to unspecific functions and processes as judged by their available Gene Ontology (GO) annotation such as binding to unfolded proteins, protein targeting, protein transport, protein tagging as well as other post transcriptional modifications other than phosphorylation, which were labeled as kinase-phophatase-related. Proteins assigned to any of the above sets and associated physical interactions were removed from the rPIN to generate a second PIN, the filtered protein-protein interaction graph (fPIN). Thus, in the fPIN, all protein interactions of proteins involved functions other than metabolism – as judged by their GO-annotation – were removed from the rPIN. It also included proteins with currently unknown function. A third PIN including only enzymes as judged by an assigned EC-number was also generated and designated as the ePIN.

Interactions with inconsistent localization annotation according to the available gene ontology, GO:Cellular-Component annotation information; i.e. interactions between proteins located in different sub-cellular compartments, were discarded from the fPIN and ePIN as well. In case of membrane-embedded proteins, interactions between proteins localized in different, but neighboring compartments were retained.

GO-Annotations for yeast genes were obtained from the SGD database [60]. The evidence codes for the gene ontology were not considered.

The GO-annotation information used to sub-set the protein data is available in the Additional File 4.

### Metabolic Interaction Networks (MINs)

Metabolic Interaction Networks (MINs) are represented in this study by Enzyme Interaction Networks (EINs) focusing on metabolic reactions as well as a Compound Interaction Networks (CIN) establishing links between metabolites directly.

The metabolic reaction lists form KEGG [61], YeastCyc [62], and the set of metabolic reactions obtained from a whole-genome metabolic reconstruction approach, in the following referred to as the Förster-Set [46], were merged and used to reconstruct the Enzyme Interaction Network (EIN). The corresponding network graph is a representation of EC-numbers and associated reactions and their metabolic interactions. Two nodes are considered connected if they share at least one common substrate or product. Ubiquitously occurring molecules, so-called currency metabolites, such as H^+^, NH_3_, H_2_O, CO_2_, and metal ions as well as coenzymes and co-substrates such as CoA, NADH+, FAD, SAM have been excluded from the analysis. In total, 51 metabolites were excluded. (see Additional File 4).

The applied connectivity conditions to generate the EIN may produce links that are theoretically possible, but that have not been experimentally verified yet. To reflect the available biological knowledge, we also generated a metabolic network from curated pathway maps, the mapEIN. For the reconstruction of the mapEIN, the relations between enzymes were extracted directly from the xml-description files of the pathway maps from the KEGG database. Two nodes in this graph are connected if both are associated with at least one common metabolite node in a map.

The EINs reflect relationships between enzymes or reactions, respectively. Alternatively, a metabolic network can be reconstructed considering metabolites themselves as nodes. Such a network, the compound (metabolite) interaction network (CIN), was constructed utilizing the reaction lists from KEGG. Two metabolites are considered connected if both are recognized as a main substrate-product reaction as annotated in KEGG, respectively. As for EINs, currency metabolites and co-enzymes and co-substrates have been discarded from consideration. The YeastCyc and Förster-Set was not considered for the construction of the CIN as both databases do not differentiate between main and side substrates or products, respectively.

### Topological properties of networks

To characterize global as well as local properties of the molecular interaction networks analyzed in this study, we computed several well established graph-theoretic network parameters.

The characteristic length (*CL*) describes the average shortest path of a graph, i.e. the expected shortest distance between any two different nodes. The *CL *was calculated applying Equation 1:

(1) $$\begin{array}{cc} CL=\frac{1}{\left|E\right|}\sum_{(i,j)\in E}d(i,j) & E=\{(i,j)\in \left\{1,2,...,N\right\}x\left\{1,2,...,N\right\}:\infty >d(i,j)\geq 0\wedge j>i\} \end{array}$$

where *d(i, j) *is the distance (shortest path) between nodes *i *and *j*, *N *is the total number of nodes, *E *defines the set of considered node pairs and |*E*| is their total number. Distances between unconnected node pairs were not considered.

The connectivity distribution, *P(k)*, was calculated according to Equation 2,

(2) $$P(k)=\frac{N_{k}}{N}$$

where *k *is the degree of nodes, i.e. the number of links associated with a node, *N *is the total number of nodes, and *N*_*k *_is the number of nodes of degree *k*. The directionality of links was not considered. Biological networks were shown to follow scale-freeness according to a power law degree distribution with *P(k) *≈ *k*^-γ^, where γ is the scale-free exponent [26-30], which was estimated by the slope of the linear regression line of degree distributions in log-log diagrams.

The cluster coefficient (*c*) is a measure of modularity of a graph. It measures to which degree the neighborhood of a node resembles a complete; i.e. fully connected graph. The cluster coefficient and its mean value were calculated according to Eqs. 3 [63].

(3) $$\begin{array}{cc} c_{i\in N_{k>1}}=\frac{\sum_{r,p\in N_{k>1}}A_{i,r}A_{i,p}A_{p,r}}{k_{i}(k_{i}-1)} & <c>=\frac{\sum_{i\in N_{k>1}}c_{i}}{\left|N_{k>1}\right|} \end{array}$$

where *A *denotes the adjacency matrix with elements set to 1 in case of an established link between nodes and zero otherwise; *k*_*i *_is the degree of node *i *for which *c *is computed, *i, p*, and *r *are indexes of all nodes in the network with k > 1.

The neighbors' connectivity *NC(k) *measures the affinity of a nodes of a particular degree to interact with nodes of either higher, similar, or lower degree. The neighbors' connectivity, *NC*, of a particular node is the average degree of its neighboring nodes [64]. *NC(k) *is the average *NC *for nodes of degree *k*. It is an increasing function of *k *when a graph is assortative, i.e. high-degree nodes preferentially tend to interact with degrees of similar, high degree. The function is decreasing when high-degree nodes preferentially interact with nodes of lower degree; then the graph is said to be dissortative. Assortativity is defined as the Pearson correlation of the degrees of neighbors, *r*_*d*_. If the distribution is uniform, *r*_*d *_equals zero, otherwise *r*_*d *_is positive for assortative graphs or negative for dissortative graphs. The assortativity was measured according to an algorithm proposed by Newman [65] (Eq. 4).

(4) $$r_{d}=\frac{{\left|E\right|}^{-1}\sum_{i\in E}j_{i}k_{i}-{\left[{\left|E\right|}^{-1}\sum_{i\in E}\frac{1}{2}\left(j_{i}^{2}+k_{i}^{2}\right)\right]}^{2}}{{\left|E\right|}^{-1}\sum_{i\in E}\frac{1}{2}\left(j_{i}^{2}+k_{i}^{2}\right)-{\left[{\left|E\right|}^{-1}\sum_{i\in E}\frac{1}{2}\left(j_{i}+k_{i}\right)\right]}^{2}}$$

where *r*_*d*_, is the assortativity, *j *and *k *are the degrees of nodes at the ends of the *i*th edge within the set of considered node pairs *E *and |*E*| is their total number, as notated for Eq. 1.

### Correlation of metabolic and protein interaction networks

The PINs were related to the EIN and mapEIN via protein-EC-number relations; i.e. proteins (enzymes) were identified in both network types and, subsequently, their pairwise distance computed in both network types. EC-number annotations were taken from KEGG [61], YeastCyc [62], SGD [60] and Expasy [66]. The relation of PINs to the CIN followed from indirect protein – EC-number-metabolite mappings according to the annotation information in KEGG. Nodes were considered equivalent in both network types, if for a metabolite (node in the CIN) the corresponding protein (nodes in the PIN) was identified via its EC-number annotation and its list of main metabolites associated with the reaction catalyzed by the enzyme.

For nodes with representation in both networks, the respective shortest distances were correlated. The distribution of distances within the PINs and MINs were evaluated by consideration of all such node couples resulting in abundance matrices. The two dimensions of the abundance matrix are the respective distances in the PINs and MINs, and the elements contain the observed counts for the respective distances pairs. Note that proteins may be assigned to more than one EC-number and can be represented multiple times in the EINs. Likewise, unique EC-numbers may be assigned to multiple proteins. The EC-numbers may comprise multiple metabolites as well. All such possible relations between the PIN and MINs were considered.

We evaluated enrichments and depletions of particular distance fields in the abundance matrix by comparing the actual counts to counts obtained from 1,000 randomly produced PIN-MIN correlations. For the randomization, protein-names within the PIN were shuffled among the graph's nodes. In this procedure, the nodes of the PINs were randomly assigned to a protein name leading to alteration of protein – EC-number relations while preserving the topology of the graphs. Statistical enrichment and depletion of actual counts versus random expectation were judged by the z-score (Equations 5) of a particular element of the abundance matrix.

(5) $$\begin{array}{l} \sigma_{{\text{rand,d}}_{\text{PIN}}{\text{,d}}_{\text{MIN}}}=\sqrt{\frac{\sum_{\text{rand}=\text{1}}^{\text{1000}}{\left(n_{{\text{rand ,d}}_{\text{PIN}}{\text{,d}}_{\text{MIN}}}-\langle n_{{\text{rand,d}}_{\text{PIN}}{\text{,d}}_{\text{MIN}}}\rangle \right)}^{2}}{\text{1000}}}\\ zScore_{{\text{d}}_{\text{PIN}}{\text{,d}}_{\text{MIN}}}=\frac{n_{{\text{observed, d}}_{\text{PIN}}{\text{, d}}_{\text{MIN}}}-\langle n_{{\text{rand,d}}_{\text{PIN}}{\text{,d}}_{\text{MIN}}}\rangle }{\sigma_{rand,{\text{d}}_{\text{PIN}}{\text{,d}}_{\text{MIN}}}}\; \end{array}$$

where *n *is the number of times a particular distance pair *d*_*PIN *_and *d*_*MIN *_was observed (*n*_*observed*_) or obtained in random networks (*n*_*rand*_), brackets indicate mean values, and *d*_*PIN *_> 0 and *d*_*MIN *_> 0 (see next paragraph).

### Treatment of multi-enzyme complexes

If subunits belonging to the same multi-enzyme complex carried identical EC numbers, their distance was considered zero and their network relationship was not analyzed further in the correlation analysis as the minimum distance included in the analyses is one. If they carried different EC numbers, their distances were computed as for any other enzyme pair given the available data.

#### The centrality of nodes

The centrality of nodes in PINs was measured either by their betweenness (*BN*) according to the algorithms proposed by Newman [67], or by the influence on the average shortest path between enzymes (*CL*_*EC*_), according to the Equations 5. While *BN *corresponds to the number of shortest paths leading through a particular node, the latter centrality measure evaluates the changes on the average shortest path length of a graph after removal of a particular node. For each node, a *z*-score of *CL*_*EC *_was calculated to judge the centrality of a node (Eqs. 6).

(6) $$\begin{array}{l} \sigma_{CL}=\sqrt{\frac{\sum_{i\in N_{EC}}{\left({\text{CL}}_{\text{EC,i}}-\langle CL_{EC}\rangle \right)}^{2}}{N_{EC}}}\\ E_{EC}=\{(i,j)\in \left\{1,2,...,N\right\}x\left\{1,2,...,N\right\}:\infty >d(i,j)\geq 0\wedge j>i\wedge i,j\in enzymes\}\\ \begin{array}{cc} CL_{EC}=\frac{1}{\left|E_{EC}\right|}\sum_{(i,j)\in E_{EC}}d(i,j) & {\text{zScore}}_{\text{i}}=\frac{\left({\text{CL}}_{\text{i}}-\langle {\text{CL}}_{\text{EC}}\rangle \right)}{\sigma_{\text{CL}}}\; \end{array} \end{array}$$

Notation as for Eq. 1.

### Correlation of PINs and metabolic flux rates

For correlating PINs and metabolic flux rates, we used flux rate data from a large-scale ^13^C-flux analysis from Blank and colleagues [47]. In this approach, flux rates of enzymes of the global metabolic network of yeast strain iFF708 [46] were estimated by flux balance analysis. In particular, we used data of flux rates measured in yeast growing in a glucose-containing medium resulting in flux data for 747 unique reactions catalyzed by 672 enzymes. The enzymes were divided into a group of enzymes with flux rates greater than 50, enzymes with flux rates between 10 and 50, flux rates of 0.1 to 1, 1.0E-4 and 0.1, and 0 to 1.0E-4 relative to an uptake of glucose set to 100 (arbitrary flux units). While the flux rates were divided according to a logarithmic scale, the range 1.0E-4 to 0.1 had been chosen to yield similar numbers of enzymes in all bins.

### Metabolic pathways

Metabolic pathways and associated proteins used in this study were taken from the SGD database. For the fatty acid synthesis pathway, malic enzyme was assumed as a source of NADPH and the malat dehydrogenase as a source of AcetylCoA and added to the pathway.

## Authors' contributions

PD and DW conceived the study, designed the analyses, interpreted the results, and wrote the manuscript. PD implemented the computational methods and carried out all computational analyses. All authors read and approved the final version of the manuscript.

## Supplementary Material

Additional file 1 **Raw counts, pairwise network (PIN, MIN) distances for Figure**3. Enrichment and depletion of the abundance of shortest path pairs between nodes represented in both the PIN and MIN. The enrichments and depletions were judged by the z-score (number in cell) of the frequency of observations (number in paranthesis) in comparison to randomized distribution with red-color indicating enrichment and blue-color depletion relative to randomized networks. Distances > 14 comprise distances greater than 14 and not connected distance pairs. Blank cells indicates 0 path pairs with z-Score of 0. Click here for file

Additional file 2 **Dependency of PIN-MIN shortest path correlations as a function of considered maximal distance**. Pearson correlation coefficient of PIN and MIN distance pairs as a function of considered maximally allowed shortest path distances in PINs and MINs. The correlation of distance pairs were calculated only including distance pairs shorter than the indicated cutoff distances d_PIN _and d_MIN_. A) rPIN and EIN; B) fPIN and EIN; C) ePIN and EIN; D) rPIN and mapEIN; E) fPIN and mapEIN; F) ePIN and mapEIN; G) rPIN and CIN; H) fPIN and CIN and I) ePIN and EIN I). Generally, a trend towards more pronounced correlations with decreasing cutoff distance is evident. Note: The drop in correlation values for short distance cutoff values is primarily explained by the inevitable loss of correlation when the considered absolute range is reduced. This is purely a statistical effect. For any correlated, but scattered data, correlation coefficients inevitably drop, if the considered range is reduced. Click here for file

Additional file 3 **Detected physical interaction of enzymes involved in selected pathways**. Detected physical interaction of enzymes involved in selected pathways. A) ergosterol biosynthesis; B) sphingolipid biosynthesis; C) ubiquinone biosynthesis; D) fatty acid biosynthesis; E) glycogen biosynthesis; F) last step of polyamine biosynthesis; G) de novo biosynthesis of pyrimidine ribonucleotides; H) superpathway of leucine, isoleucine, and valine biosynthesis; I) lysine biosynthesis; J) superpathway of phenylalanine, tyrosine and tryptophan biosynthesis. In picture A) HMG1/2, MVD1, IDI1 and ERG10/13 are part of the mevalonate pathway. All pathways are derived from the SGD Database. Only enzymes contained in the PIN are visualized, i.e. the pathways are not complete in a biochemical sense. For the fatty acids biosynthesis, the malic enzymes (MAE) as well as the malate dehydrogenase (MDH2) were included as sources of NADPH and AcetylCoA. Enzymes are abbreviated by their gene symbols and detected interactions between them are denoted by connecting lines. Click here for file

Additional file 4 **Supplementary Annotation and Raw Count Data. GO categories used for creating filtered version of protein interaction networks**; List of all 51 molecules (currency metabolites, co-factors) excluded from the analysis. Click here for file
