# Supplementary figures and images for: The integrated analysis of metabolic and protein interaction networks reveals novel molecular organizing principles
Source: BMC Syst Biol. 2008 Nov 25;2:100. doi: 10.1186/1752-0509-2-100 (PMC2607255; doi:10.1186/1752-0509-2-100)

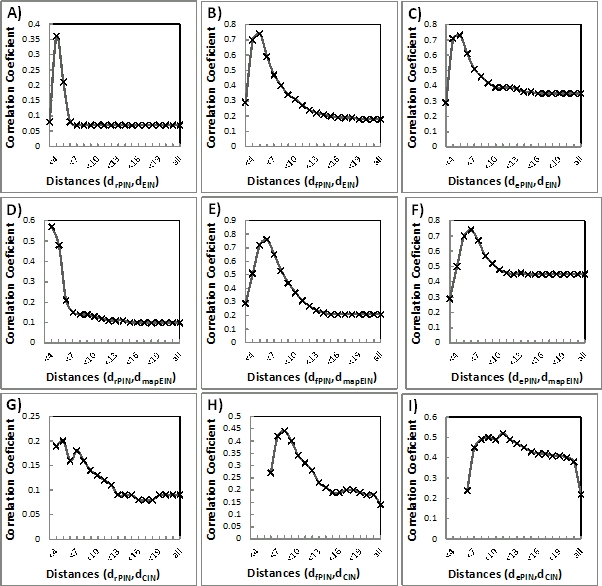

Supplement: Additional file 2 — Dependency of PIN-MIN shortest path correlations as a function of considered maximal distance. Pearson correlation coefficient of PIN and MIN distance pairs as a function of considered maximally allowed shortest path distances in PINs and MINs. The correlation of distance pairs were calculated only including distance pairs shorter than the indicated cutoff distances dPIN and dMIN. A) rPIN and EIN; B) fPIN and EIN; C) ePIN and EIN; D) rPIN and mapEIN; E) fPIN and mapEIN; F) ePIN and mapEIN; G) rPIN and CIN; H) fPIN and CIN and I) ePIN and EIN I). Generally, a trend towards more pronounced correlations with decreasing cutoff distance is evident. Note: The drop in correlation values for short distance cutoff values is primarily explained by the inevitable loss of correlation when the considered absolute range is reduced. This is purely a statistical effect. For any correlated, but scattered data, correlation coefficients inevitably drop, if the considered range is reduced. [file 1752-0509-2-100-S2.jpeg]

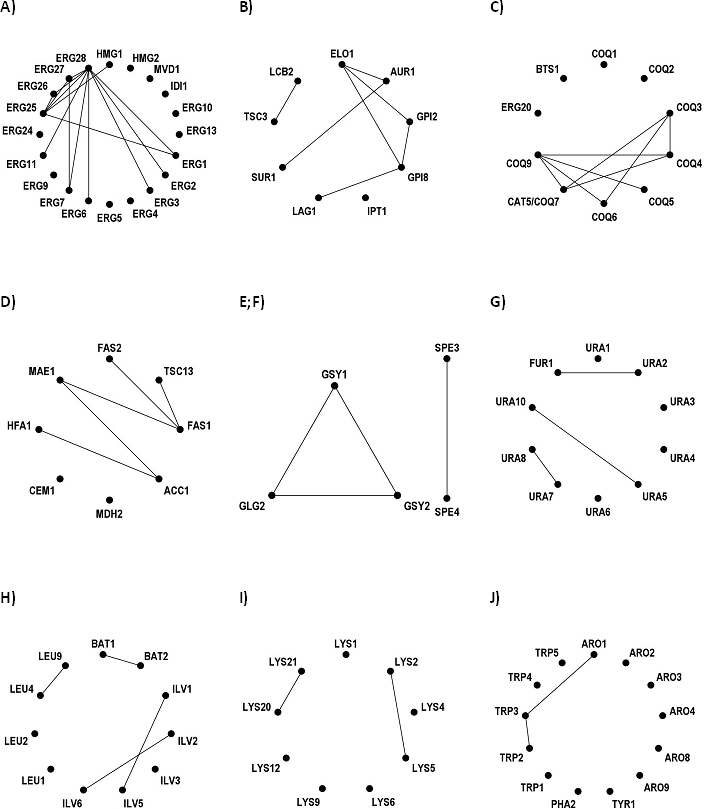

Supplement: Additional file 3 — Detected physical interaction of enzymes involved in selected pathways. Detected physical interaction of enzymes involved in selected pathways. A) ergosterol biosynthesis; B) sphingolipid biosynthesis; C) ubiquinone biosynthesis; D) fatty acid biosynthesis; E) glycogen biosynthesis; F) last step of polyamine biosynthesis; G) de novo biosynthesis of pyrimidine ribonucleotides; H) superpathway of leucine, isoleucine, and valine biosynthesis; I) lysine biosynthesis; J) superpathway of phenylalanine, tyrosine and tryptophan biosynthesis. In picture A) HMG1/2, MVD1, IDI1 and ERG10/13 are part of the mevalonate pathway. All pathways are derived from the SGD Database. Only enzymes contained in the PIN are visualized, i.e. the pathways are not complete in a biochemical sense. For the fatty acids biosynthesis, the malic enzymes (MAE) as well as the malate dehydrogenase (MDH2) were included as sources of NADPH and AcetylCoA. Enzymes are abbreviated by their gene symbols and detected interactions between them are denoted by connecting lines. [file 1752-0509-2-100-S3.jpeg]
