# Supplementary material for: The integrated analysis of metabolic and protein interaction networks reveals novel molecular organizing principles
Source: BMC Syst Biol. 2008 Nov 25;2:100. doi: 10.1186/1752-0509-2-100 (PMC2607255; doi:10.1186/1752-0509-2-100)
Supplement: Additional file 4 — Supplementary Annotation and Raw Count Data. GO categories used for creating filtered version of protein interaction networks; List of all 51 molecules (currency metabolites, co-factors) excluded from the analysis. [file 1752-0509-2-100-S4.pdf]

GO Annotations used for identifying Protein Degradation/ Ubiquitin associated proteins

GO:0000151	ubiquitin ligase complex
GO:0001509	legumain activity
GO:0004175	endopeptidase activity
GO:0004176	ATP-dependent peptidase activity
GO:0004177	aminopeptidase activity
GO:0004178	leucyl aminopeptidase activity
GO:0004179	membrane alanyl aminopeptidase activity
GO:0004180	carboxypeptidase activity
GO:0004182	carboxypeptidase A activity
GO:0004185	serine carboxypeptidase activity
GO:0004186	carboxypeptidase C activity
GO:0004187	carboxypeptidase D activity
GO:0004190	aspartic-type endopeptidase activity
GO:0004191	barrierpepsin activity
GO:0004194	pepsin A activity
GO:0004196	saccharopepsin activity
GO:0004197	cysteine-type endopeptidase activity
GO:0004198	calpain activity
GO:0004221	ubiquitin thiolesterase activity
GO:0004222	metalloendopeptidase activity
GO:0004226	Gly-X carboxypeptidase activity
GO:0004239	methionyl aminopeptidase activity
GO:0004240	mitochondrial processing peptidase activity
GO:0004243	mitochondrial intermediate peptidase activity
GO:0004244	mitochondrial inner membrane peptidase activity
GO:0004247	saccharolysin activity
GO:0004250	aminopeptidase I activity
GO:0004252	serine-type endopeptidase activity
GO:0004262	cerevisin activity
GO:0004274	dipeptidyl-peptidase IV activity
GO:0004287	prolyl oligopeptidase activity
GO:0004289	subtilase activity
GO:0004298	threonine endopeptidase activity
GO:0004839	ubiquitin activating enzyme activity
GO:0004839	ubiquitin activating enzyme activity
GO:0004842	ubiquitin conjugating enzyme activity
GO:0004843	ubiquitin-specific protease activity
GO:0004866	endopeptidase inhibitor activity
GO:0004867	serine-type endopeptidase inhibitor activity
GO:0005680	anaphase-promoting complex
GO:0006511	ubiquitin-dependent protein catabolic process
GO:0006512	ubiquitin cycle
GO:0008054	cyclin catabolic process
GO:0008233	peptidase activity
GO:0008234	cysteine-type peptidase activity
GO:0008235	metalloexopeptidase activity
GO:0008236	serine-type peptidase activity
GO:0008237	metallopeptidase activity
GO:0008423	bleomycin hydrolase activity
GO:0008450	O-sialoglycoprotein endopeptidase activity
GO:0008451	X-Pro aminopeptidase activity
GO:0008487	prenyl-dependent CAAX protease activity
GO:0008641	small protein activating enzyme activity
GO:0008717	D-alanyl-D-alanine endopeptidase activity

GO:0008769	X-His dipeptidase activity
GO:0009003	signal peptidase activity
GO:0009049	aspartic-type signal peptidase activity
GO:0016574	histone ubiquitination
GO:0016806	dipeptidyl-peptidase and tripeptidyl-peptidase activity
GO:0016929	SUMO-specific protease activity
GO:0017039	dipeptidyl-peptidase III activity
GO:0019005	SCF ubiquitin ligase complex
GO:0019778	APG12 activating enzyme activity
GO:0019779	APG8 activating enzyme activity
GO:0019781	NEDD8 activating enzyme activity
GO:0019787	small conjugating protein ligase activity
GO:0019789	SUMO ligase activity
GO:0019948	SUMO activating enzyme activity
GO:0030414	protease inhibitor activity
GO:0030433	ER-associated protein catabolic process
GO:0030693	caspase activity
GO:0031145	anaphase-promoting complex-dependent proteasomal ubiquitin-dependent protein catabolic process
GO:0031146	SCF-dependent proteasomal ubiquitin-dependent protein catabolic process
GO:0031371	ubiquitin conjugating enzyme complex
GO:0031386	protein tag
GO:0031463	Cul3-RING ubiquitin ligase complex
GO:0032435	negative regulation of proteasomal ubiquitin-dependent protein catabolic process
GO:0042292	URM1 activating enzyme activity
GO:0042576	aspartyl aminopeptidase activity
GO:0042787	protein ubiquitination during ubiquitin-dependent protein catabolic process
GO:0043130	ubiquitin binding
GO:0043161	proteasomal ubiquitin-dependent protein catabolic process
GO:0043162	ubiquitin-dependent protein catabolic process via the multivesicular body pathway
GO:0043224	nuclear SCF ubiquitin ligase complex
GO:0043328	protein targeting to vacuole during ubiquitin-dependent protein catabolic process via the MVB pathway
GO:0051436	negative regulation of ubiquitin ligase activity during mitotic cell cycle
GO:0051443	positive regulation of ubiquitin ligase activity

GO Annotations used for identifying Kinase/Phosphatase proteins

GO:0000155	two-component sensor activity
GO:0000156	two-component response regulator activity
GO:0000158	protein phosphatase type 2A activity
GO:0000159	protein phosphatase type 2A complex
GO:0000163	protein phosphatase type 1 activity
GO:0000164	protein phosphatase type 1 complex
GO:0003924	GTPase activity
GO:0004672	protein kinase activity
GO:0004673	protein histidine kinase activity
GO:0004674	protein serine/threonine kinase activity
GO:0004679	AMP-activated protein kinase activity
GO:0004680	casein kinase activity
GO:0004681	casein kinase I activity
GO:0004682	protein kinase CK2 activity
GO:0004683	calmodulin regulated protein kinase activity
GO:0004684	calmodulin-dependent protein kinase I activity
GO:0004685	calcium- and calmodulin-dependent protein kinase activity
GO:0004691	cAMP-dependent protein kinase activity
GO:0004693	cyclin-dependent protein kinase activity
GO:0004694	eukaryotic translation initiation factor 2alpha kinase activity
GO:0004696	glycogen synthase kinase 3 activity
GO:0004697	protein kinase C activity
GO:0004702	receptor signaling protein serine/threonine kinase activity
GO:0004707	MAP kinase activity
GO:0004708	MAP kinase kinase activity
GO:0004709	MAP kinase kinase kinase activity
GO:0004712	protein threonine/tyrosine kinase activity
GO:0004713	protein-tyrosine kinase activity
GO:0004721	phosphoprotein phosphatase activity
GO:0004722	protein serine/threonine phosphatase activity
GO:0004723	calcium-dependent protein serine/threonine phosphatase activity
GO:0004725	protein tyrosine phosphatase activity
GO:0004727	prenylated protein tyrosine phosphatase activity
GO:0004860	protein kinase inhibitor activity
GO:0004861	cyclin-dependent protein kinase inhibitor activity
GO:0004862	cAMP-dependent protein kinase inhibitor activity
GO:0004864	protein phosphatase inhibitor activity
GO:0004871	signal transducer activity
GO:0004872	receptor activity
GO:0004888	transmembrane receptor activity
GO:0004930	G-protein coupled receptor activity
GO:0004932	mating-type factor pheromone receptor activity
GO:0004933	mating-type a-factor pheromone receptor activity
GO:0004934	mating-type alpha-factor pheromone receptor activity
GO:0005034	osmosensor activity
GO:0005057	receptor signaling protein activity
GO:0005083	small GTPase regulator activity
GO:0005955	calcineurin complex
GO:0008138	protein tyrosine/serine/threonine phosphatase activity
GO:0008158	hedgehog receptor activity
GO:0008287	protein serine/threonine phosphatase complex

GO:0008330	protein tyrosine/threonine phosphatase activity
GO:0008349	MAP kinase kinase kinase kinase activity
GO:0008597	calcium-dependent protein serine/threonine phosphatase regulator activity
GO:0008599	protein phosphatase type 1 regulator activity
GO:0008601	protein phosphatase type 2A regulator activity
GO:0008603	cAMP-dependent protein kinase regulator activity
GO:0008605	protein kinase CK2 regulator activity
GO:0015071	protein phosphatase type 2C activity
GO:0016299	regulator of G-protein signaling activity
GO:0016301	kinase activity
GO:0016538	cyclin-dependent protein kinase regulator activity
GO:0017017	MAP kinase phosphatase activity
GO:0019207	kinase regulator activity
GO:0019209	kinase activator activity
GO:0019211	phosphatase activator activity
GO:0019828	aspartic-type endopeptidase inhibitor activity
GO:0019887	protein kinase regulator activity
GO:0019888	protein phosphatase regulator activity
GO:0019912	cyclin-dependent protein kinase activating kinase activity
GO:0030295	protein kinase activator activity
GO:0030695	GTPase regulator activity
GO:0033550	MAP kinase tyrosine phosphatase activity
GO:0035174	histone serine kinase activity
GO:0043539	protein serine/threonine kinase activator activity

GO Annotations used for identifying DNA-related proteins

GO:0000014	single-stranded DNA specific endodeoxyribonuclease activity
GO:0000049	tRNA binding
GO:0000124	SAGA complex
GO:0000149	SNARE binding
GO:0000175	3'-5'-exoribonuclease activity
GO:0000179	rRNA (adenine-N6,N6-)-dimethyltransferase activity
GO:0000182	rDNA binding
GO:0000213	tRNA-intron endonuclease activity
GO:0000215	tRNA 2'-phosphotransferase activity
GO:0000339	RNA cap binding
GO:0000400	four-way junction DNA binding
GO:0000403	Y-form DNA binding
GO:0003677	DNA binding
GO:0003678	DNA helicase activity
GO:0003680	AT DNA binding
GO:0003682	chromatin binding
GO:0003684	damaged DNA binding
GO:0003688	DNA replication origin binding
GO:0003689	DNA clamp loader activity
GO:0003690	double-stranded DNA binding
GO:0003697	single-stranded DNA binding
GO:0003700	transcription factor activity
GO:0003701	RNA polymerase I transcription factor activity
GO:0003702	RNA polymerase II transcription factor activity
GO:0003704	specific RNA polymerase II transcription factor activity
GO:0003706	ligand-regulated transcription factor activity
GO:0003709	RNA polymerase III transcription factor activity
GO:0003711	transcriptional elongation regulator activity
GO:0003712	transcription cofactor activity
GO:0003713	transcription coactivator activity
GO:0003714	transcription corepressor activity
GO:0003723	RNA binding
GO:0003724	RNA helicase activity
GO:0003729	mRNA binding
GO:0003735	structural constituent of ribosome
GO:0003743	translation initiation factor activity
GO:0003746	translation elongation factor activity
GO:0003747	translation release factor activity
GO:0003887	DNA-directed DNA polymerase activity
GO:0003889	alpha DNA polymerase activity
GO:0003890	beta DNA polymerase activity
GO:0003891	delta DNA polymerase activity
GO:0003893	epsilon DNA polymerase activity
GO:0003894	zeta DNA polymerase activity
GO:0003895	gamma DNA-directed DNA polymerase activity
GO:0003896	DNA primase activity
GO:0003899	DNA-directed RNA polymerase activity
GO:0003905	alkylbase DNA N-glycosylase activity
GO:0003906	DNA-(apurinic or apyrimidinic site) lyase activity
GO:0003908	methylated-DNA-[protein]-cysteine S-methyltransferase activity
GO:0003910	DNA ligase (ATP) activity
GO:0003917	DNA topoisomerase type I activity

GO:0003918	DNA topoisomerase (ATP-hydrolyzing) activity
GO:0004003	ATP-dependent DNA helicase activity
GO:0004004	ATP-dependent RNA helicase activity
GO:0004045	aminoacyl-tRNA hydrolase activity
GO:0004402	histone acetyltransferase activity
GO:0004406	H3/H4 histone acetyltransferase activity
GO:0004407	histone deacetylase activity
GO:0004479	methionyl-tRNA formyltransferase activity
GO:0004482	mRNA (guanine-N7-)-methyltransferase activity
GO:0004484	mRNA guanylyltransferase activity
GO:0004808	tRNA (5-methylaminomethyl-2-thiouridylate)-methyltransferase activity
GO:0004809	tRNA (guanine-N2-)-methyltransferase activity
GO:0004810	tRNA adenylyltransferase activity
GO:0004811	tRNA isopentenyltransferase activity
GO:0004813	alanine-tRNA ligase activity
GO:0004814	arginine-tRNA ligase activity
GO:0004815	aspartate-tRNA ligase activity
GO:0004816	asparagine-tRNA ligase activity
GO:0004817	cysteine-tRNA ligase activity
GO:0004818	glutamate-tRNA ligase activity
GO:0004819	glutamine-tRNA ligase activity
GO:0004820	glycine-tRNA ligase activity
GO:0004821	histidine-tRNA ligase activity
GO:0004822	isoleucine-tRNA ligase activity
GO:0004823	leucine-tRNA ligase activity
GO:0004824	lysine-tRNA ligase activity
GO:0004825	methionine-tRNA ligase activity
GO:0004826	phenylalanine-tRNA ligase activity
GO:0004827	proline-tRNA ligase activity
GO:0004828	serine-tRNA ligase activity
GO:0004829	threonine-tRNA ligase activity
GO:0004830	tryptophan-tRNA ligase activity
GO:0004831	tyrosine-tRNA ligase activity
GO:0004832	valine-tRNA ligase activity
GO:0004844	uracil DNA N-glycosylase activity
GO:0005671	Ada2/Gcn5/Ada3 transcription activator complex
GO:0006608	snRNP protein import into nucleus
GO:0006609	mRNA-binding (hnRNP) protein import into nucleus
GO:0008079	translation termination factor activity
GO:0008094	DNA-dependent ATPase activity
GO:0008134	transcription factor binding
GO:0008135	translation factor activity, nucleic acid binding
GO:0008159	positive transcription elongation factor activity
GO:0008173	RNA methyltransferase activity
GO:0008174	mRNA methyltransferase activity
GO:0008175	tRNA methyltransferase activity
GO:0008193	tRNA guanylyltransferase activity
GO:0008251	tRNA specific adenosine deaminase activity
GO:0008301	DNA bending activity
GO:0008419	RNA lariat debranching enzyme activity
GO:0008534	oxidized purine base lesion DNA N-glycosylase activity
GO:0008650	rRNA (uridine-2'-O-)-methyltransferase activity
GO:0008989	rRNA (guanine-N1-)-methyltransferase activity
GO:0010390	histone monoubiquitination
GO:0015999	eta DNA polymerase activity
GO:0016149	translation release factor activity, codon specific

GO:0016251	general RNA polymerase II transcription factor activity
GO:0016423	tRNA (guanine) methyltransferase activity
GO:0016424	tRNA (guanosine) methyltransferase activity
GO:0016428	tRNA (cytosine-5-)-methyltransferase activity
GO:0016429	tRNA (adenine-N1-)-methyltransferase activity
GO:0016431	tRNA (uridine) methyltransferase activity
GO:0016439	tRNA-pseudouridine synthase activity
GO:0016455	RNA polymerase II transcription mediator activity
GO:0016563	transcriptional activator activity
GO:0016564	transcriptional repressor activity
GO:0016565	general transcriptional repressor activity
GO:0016566	specific transcriptional repressor activity
GO:0016944	RNA polymerase II transcription elongation factor activity
GO:0017005	tyrosyl-DNA phosphodiesterase activity
GO:0017136	NAD-dependent histone deacetylase activity
GO:0017150	tRNA dihydrouridine synthase activity
GO:0019237	centromeric DNA binding
GO:0019843	rRNA binding
GO:0030188	chaperone regulator activity
GO:0030337	DNA polymerase processivity factor activity
GO:0030371	translation repressor activity
GO:0030515	snoRNA binding
GO:0030528	transcription regulator activity
GO:0030620	U2 snRNA binding
GO:0031202	RNA splicing factor activity, transesterification mechanism
GO:0031490	chromatin DNA binding
GO:0032041	NAD-dependent histone deacetylase activity (H3-K14 specific)
GO:0032777	Piccolo NuA4 histone acetyltransferase complex
GO:0033100	NuA3 histone acetyltransferase complex
GO:0035267	NuA4 histone acetyltransferase complex
GO:0042054	histone methyltransferase activity
GO:0042134	rRNA primary transcript binding
GO:0042162	telomeric DNA binding
GO:0042393	histone binding
GO:0042800	histone lysine N-methyltransferase activity (H3-K4 specific)
GO:0043140	ATP-dependent 3' to 5' DNA helicase activity
GO:0043141	ATP-dependent 5' to 3' DNA helicase activity
GO:0043166	H4/H2A histone acetyltransferase activity
GO:0043189	H4/H2A histone acetyltransferase complex
GO:0045129	NAD-independent histone deacetylase activity
GO:0045182	translation regulator activity
GO:0046695	SLIK (SAGA-like) complex
GO:0046969	NAD-dependent histone deacetylase activity (H3-K9 specific)
GO:0046970	NAD-dependent histone deacetylase activity (H4-K16 specific)
GO:0050072	m7G(5')pppN diphosphatase activity
GO:0051500	D-tyrosyl-tRNA(Tyr) deacylase activity
GO:0051864	histone demethylase activity (H3-K36 specific)

GO Annotations used for identifying other, non-metabolic proteins

GO:0000054	ribosome export from nucleus
GO:0000055	ribosomal large subunit export from nucleus
GO:0000056	ribosomal small subunit export from nucleus
GO:0000059	protein import into nucleus, docking
GO:0000060	protein import into nucleus, translocation
GO:0000149	SNARE binding
GO:0000208	nuclear translocation of MAPK during osmolarity sensing
GO:0000268	peroxisome targeting sequence binding
GO:0000290	deadenylation-dependent decapping
GO:0001671	ATPase stimulator activity
GO:0003923	GPI-anchor transamidase activity
GO:0003924	GTPase activity
GO:0004175	endopeptidase activity
GO:0004596	peptide alpha-N-acetyltransferase activity
GO:0004857	enzyme inhibitor activity
GO:0004860	protein kinase inhibitor activity
GO:0004871	signal transducer activity
GO:0005084	Rab escort protein activity
GO:0005085	guanyl-nucleotide exchange factor activity
GO:0005086	ARF guanyl-nucleotide exchange factor activity
GO:0005088	Ras guanyl-nucleotide exchange factor activity
GO:0005089	Rho guanyl-nucleotide exchange factor activity
GO:0005093	Rab GDP-dissociation inhibitor activity
GO:0005094	Rho GDP-dissociation inhibitor activity
GO:0005095	GTPase inhibitor activity
GO:0005096	GTPase activator activity
GO:0005097	Rab GTPase activator activity
GO:0005098	Ran GTPase activator activity
GO:0005099	Ras GTPase activator activity
GO:0005100	Rho GTPase activator activity
GO:0006605	protein targeting
GO:0006606	protein import into nucleus
GO:0006607	NLS-bearing substrate import into nucleus
GO:0006610	ribosomal protein import into nucleus
GO:0006611	protein export from nucleus
GO:0006612	protein targeting to membrane
GO:0006613	cotranslational protein targeting to membrane
GO:0006614	SRP-dependent cotranslational protein targeting to membrane
GO:0006616	SRP-dependent cotranslational protein targeting to membrane,
GO:0006617	SRP-dependent cotranslational protein targeting to membrane, signal cognition
GO:0006620	posttranslational protein targeting to membrane
GO:0006623	protein targeting to vacuole
GO:0006625	protein targeting to peroxisome
GO:0006626	protein targeting to mitochondrion
GO:0006627	mitochondrial protein processing
GO:0006886	intracellular protein transport
GO:0007092	anaphase-promoting complex activation during mitotic cell cycle
GO:0008047	enzyme activator activity
GO:0008060	ARF GTPase activator activity
GO:0008139	nuclear localization sequence binding

GO:0008308	voltage-gated ion-selective channel activity
GO:0008320	protein carrier activity
GO:0008538	proteasome activator activity
GO:0008565	protein transporter activity
GO:0015664	nicotinamide mononucleotide permease activity
GO:0016538	cyclin-dependent protein kinase regulator activity
GO:0016558	protein import into peroxisome matrix
GO:0016560	protein import into peroxisome matrix, docking
GO:0016562	protein import into peroxisome matrix, receptor recycling
GO:0016598	protein arginylation
GO:0016925	protein sumoylation
GO:0017112	Rab guanyl-nucleotide exchange factor activity
GO:0019211	phosphatase activator activity
GO:0030150	protein import into mitochondrial matrix
GO:0030189	chaperone activator activity
GO:0030190	chaperone inhibitor activity
GO:0030234	enzyme regulator activity
GO:0030970	retrograde protein transport, ER to cytosol
GO:0031204	posttranslational protein targeting to membrane, translocation
GO:0031386	protein tag
GO:0032258	CVT pathway
GO:0032527	protein exit from endoplasmic reticulum
GO:0042719	mitochondrial intermembrane space protein transporter complex
GO:0042992	negative regulation of transcription factor import into nucleus
GO:0042994	cytoplasmic sequestering of transcription factor
GO:0043001	Golgi to plasma membrane protein transport
GO:0043328	protein targeting to vacuole during ubiquitin-dependent protein catabolic he MVB pathway
GO:0045039	protein import into mitochondrial inner membrane
GO:0045040	protein import into mitochondrial outer membrane
GO:0045041	protein import into mitochondrial intermembrane space
GO:0045046	protein import into peroxisome membrane
GO:0045047	protein targeting to ER
GO:0048306	calcium-dependent protein binding
GO:0051082	unfolded protein binding

currency metabolites, co-factors removed from the metabolic network

C00001	H2o
C00002	ATP
C00003	NAD+
C00004	NADH
C00005	NADPH
C00006	NADP+
C00007	Oxygen;O2
C00008	ADP
C00009	Orthophosphate,Pi
C00010	CoA
C00011	CO2
C00013	Pyrophosphate;PPi
C00014	NH3
C00015	UDP
C00016	FAD
C00018	Pyridoxalphosphate
C00019	S-Adenosyl-L-methionine; SAM
C00020	AMP
C00021	S-Adenosylhomocysteine; SAH
C00023	Iron
C00027	H2O2
C00028	Acceptor
C00030	Reduced acceptor
C00034	Manganese
C00035	GDP
C00038	Zinc
C00044	GTP
C00050	Metal
C00055	CMP
C00061	FMN
C00063	CTP
C00070	Copper
C00075	UTP
C00076	Calcium;Ca2+
C00080	H+
C00105	UMP
C00112	CDP
C00113	PQQ
C00115	Chloride
C00120	Biotin;D-Biotin;VitaminH;CoenzymeR
C00125	Ferricytochromec;Cytochromec3+
C00126	Ferrocytochromec;Cytochromec2+;Reducedcytochromec
C00138	Reducedferredoxin
C00139	Oxidizedferredoxin
C00144	GMP
C00175	Cobalt;Co2+
C00194	Cobamidecoenzyme
C00205	hn;Light
C00238	Potassium;K+
C00291	Nickel;Ni2+
C01352	FADH2
